# Photosensitizing Lipid Nanoparticles for Ferroptosis‐Enhanced Photodynamic Cancer Therapy via GPX4 Silencing

**DOI:** 10.1002/adhm.202503748

**Published:** 2025-11-12

**Authors:** Ga‐Hyun Bae, Seungyong Shin, Joo Dong Park, Eun‐Young Koh, Seunghyo Ko, Jieun Han, Chun Gown Park, Dong‐Hyun Kim, Kun Na, Wooram Park

**Affiliations:** ^1^ Department of MetaBioHealth Institute for Cross‐disciplinary Studies (ICS) Sungkyunkwan University (SKKU) Seobu‐ro 2066 Suwon Gyeonggi 16419 Republic of Korea; ^2^ Department of Integrative Biotechnology College of Biotechnology and Bioengineering, SKKU Seobu‐ro 2066 Suwon Gyeonggi 16419 Republic of Korea; ^3^ Department of Biomedical Engineering ICS, SKKU Seobu‐ro 2066 Suwon Gyeonggi 16419 Republic of Korea; ^4^ Department of Radiology Feinberg School of Medicine Northwestern University Chicago IL 60611 USA; ^5^ Robert H. Lurie Comprehensive Cancer Center Northwestern University Chicago IL 60611 USA; ^6^ Department of Biomedical Engineering McCormick School of Engineering Northwestern University Evanston IL 60208 USA; ^7^ Department of Biomedical‐Chemical Engineering The Catholic University of Korea Jibong‐ro 43 Bucheon Gyeonggi 14662 Republic of Korea; ^8^ Department of Biotechnology The Catholic University of Korea Jibong‐ro 43 Bucheon Gyeonggi 14662 Republic of Korea

**Keywords:** cancer nanomedicine, ferroptosis, GPX4 silencing, lipid nanoparticles, photodynamic therapy

## Abstract

Ferroptosis, a regulated form of non‐apoptotic cell death driven by iron‐dependent lipid peroxidation, has emerged as a promising approach for overcoming therapy‐resistant cancers. A multifunctional lipid nanoparticle (LNP) platform was developed to integrate ferroptosis induction with photodynamic therapy (PDT) for synergistic anticancer effects. By partially substituting cholesterol in conventional DLin‐MC3‐DMA (MC3)‐based LNPs with cholesterol—polyethylene glycol (PEG)–pheophorbide a (CPP), we engineered photosensitizing lipid nanoparticles (PLNPs) capable of delivering glutathione peroxidase 4 (GPX4)‐targeting small interfering RNA (siRNA). Upon laser irradiation, the PLNPs generate reactive oxygen species (ROS) through PDT, while siRNA‐mediated GPX4 silencing promotes ferroptosis by disrupting cellular antioxidant defenses. The PLNPs demonstrate favorable physicochemical characteristics, efficient gene silencing, and potent ROS production. In vitro experiments in 4T1 and EO771 breast cancer cells reveal enhanced cytotoxicity under combined treatment, underscoring the synergistic interaction between PDT‐induced oxidative stress and ferroptotic cell death. In vivo, the PLNPs exhibit prolonged tumor retention, effective GPX4 knockdown, and significant tumor growth inhibition, with minimal systemic toxicity. Overall, this work introduces a dual‐function nanoplatform that potentiates photodynamic cancer therapy through ferroptosis induction and offers a versatile strategy for developing next‐generation combination treatments targeting aggressive tumors.

## Graphical abstract

Photosensitizing lipid nanoparticles (PLNPs) are engineered by incorporating cholesterol–PEG–pheophorbide a into MC3‐based LNPs and encapsulating GPX4‐targeting siRNA. Upon light activation, PLNPs generate reactive oxygen species (ROS) while silencing GPX4 to induce ferroptosis. This dual‐action approach—combining photodynamic therapy with ferroptotic cell death—significantly enhances anticancer efficacy, highlighting the therapeutic potential of this dual‐action nanoplatform for ferroptosis‐augmented cancer therapy.

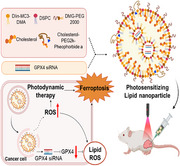

## 1 Introduction

Despite significant advancements in chemotherapy, radiotherapy, and targeted therapies, many malignant tumors remain difficult to eradicate due to intrinsic or acquired resistance and the complex nature of the tumor microenvironment.^[^ ^1^, ^2^ ^]^ Consequently, there is growing interest in alternative therapeutic strategies that bypass traditional apoptotic pathways and instead exploit non‐apoptotic forms of regulated cell death. Among these, ferroptosis—a regulated form of cell death driven by iron (Fe)‐dependent lipid peroxidation—has attracted considerable attention as a promising anticancer strategy.^[^ ^3^, ^4^, ^5^ ^]^

Glutathione peroxidase 4 (GPX4) is a key enzyme that utilizes glutathione (GSH) as a cofactor to reduce lipid hydroperoxides to non‐toxic phospholipid alcohols, thereby suppressing ferroptotic cell death.^[^ ^6^, ^7^ ^]^ Inhibition or knockdown of GPX4 increases intracellular oxidative stress and effectively induces ferroptosis in cancer cells.^[^ ^8^, ^9^ ^]^ Notably, small interfering RNA (siRNA)‐mediated silencing of GPX4 has demonstrated effective ferroptosis induction in various tumor models.^[^ ^10^ ^]^ However, clinical application of this approach remains limited, primarily due to challenges in achieving efficient in vivo delivery and spatiotemporal control of siRNA activity.^[^ ^11^, ^12^, ^13^ ^]^

Lipid nanoparticles (LNPs) have emerged as clinically validated platforms for siRNA delivery. DLin‐MC3‐DMA (MC3)‐based LNPs serve as the foundation for the FDA‐approved siRNA drug Onpattro.^[^ ^14^, ^15^, ^16^ ^]^ These nanoparticles—typically composed of MC3, DSPC, cholesterol, and polyethylene glycol (PEG)‐lipids—offer excellent protection for siRNA, high intracellular delivery efficiency, and low toxicity.^[^ ^11^, ^17^ ^]^ Moreover, recent advances in nanomedicine‐based drug delivery systems have further expanded the therapeutic potential of LNPs, particularly in cancer treatment.^[^ ^18^, ^19^ ^]^ Nonetheless, conventional LNPs mainly act as passive delivery vehicles, and their therapeutic efficacy depends entirely on the functionality of the encapsulated siRNA.

Photodynamic therapy (PDT), a clinically established cancer treatment, involves the use of a photosensitizer,^[^ ^20^ ^]^ light of a specific wavelength, and molecular oxygen to generate reactive oxygen species (ROS), ultimately leading to cell death.^[^ ^21^, ^22^, ^23^ ^]^ PDT offers several advantages, including localized treatment, minimal invasiveness, and high selectivity for tumor tissues, which have facilitated its clinical adoption.^[^ ^24^, ^25^ ^]^ Interestingly, PDT‐induced oxidative stress has been shown to engage with the ferroptosis pathway, suggesting the potential for synergistic therapeutic effects when these modalities are combined.^[^ ^26^, ^27^, ^28^, ^29^ ^]^

**Scheme 1:**
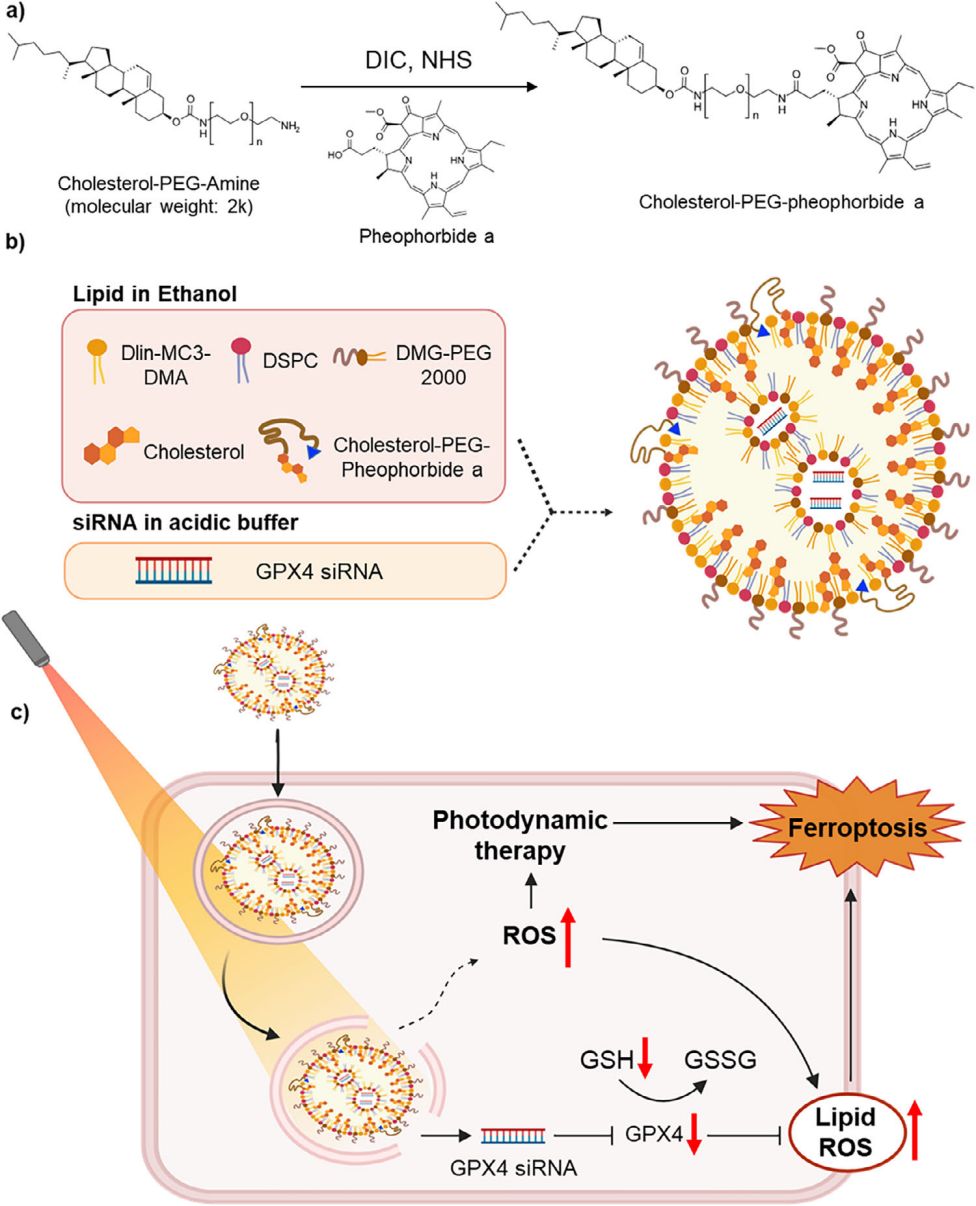
Schematic illustration of a ferroptosis‐inducing cancer treatment strategy using photosensitizing lipid nanoparticles (PLNPs). a) Synthesis of cholesterol–PEG–pheophorbide a (CPP) via DIC/NHS‐mediated conjugation. b) Formulation of PLNPs incorporating CPP and siGPX4 through ethanol injection and citrate buffer mixing. c) Upon laser irradiation, siGPX4‐loaded PLNPs synergistically induce ferroptosis via GPX4 gene silencing and photodynamic therapy (PDT).

In this study, we propose a dual‐mode cancer therapy strategy that integrates GPX4‐targeted ferroptosis with PDT‐induced oxidative stress. Specifically, we developed photosensitizing lipid nanoparticles (PLNPs) by partially replacing cholesterol with cholesterol– PEG–pheophorbide a (CPP), a PEGylated photosensitizer capable of generating ROS upon light irradiation (Scheme 1a). This modification preserves the favorable delivery characteristics of conventional LNPs while enabling ROS generation in response to external light stimulation. By encapsulating GPX4‐targeting siRNA within this platform, we aim to induce ferroptosis synergistically through both gene silencing and PDT‐mediated oxidative stress (Scheme 1b,c).^[^ ^10^, ^30^ ^]^

We systematically evaluated the physicochemical properties, gene silencing efficiency, photoreactivity, and in vitro cytotoxicity of the PLNPs in cancer cell models. Additionally, we investigated the underlying mechanisms of ferroptosis induction and the potential synergy between the two therapeutic modalities. Collectively, our findings demonstrate the potential of this dual‐functional nanoplatform as a next‐generation strategy for combination cancer therapy.

## 2 Results and Discussion

### 2.1 Synthesis of Cholesterol–PEG–Pheophorbide a (CPP) and Characterization of Photosensitizing Lipid Nanoparticles (PLNPs)

**Figure 1:**
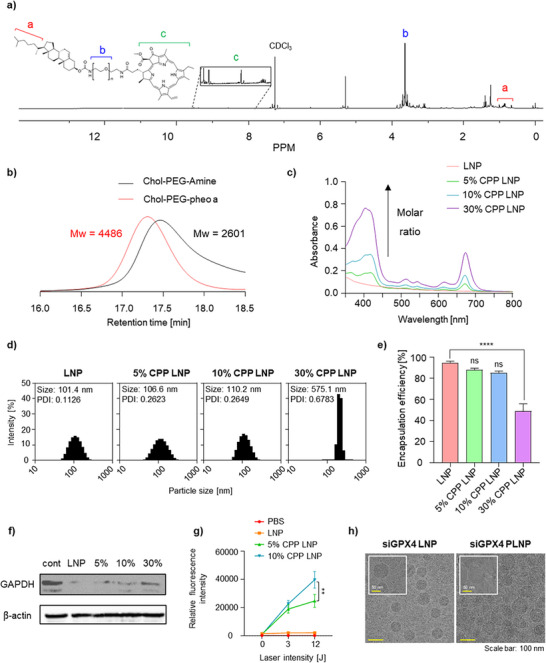
Synthesis of cholesterol–PEG–pheophorbide a (CPP) and characterization of photosensitizing lipid nanoparticles (PLNPs). a) ^−1^H NMR (300 MHz) spectrum of CPP in CDCl_3_. b) Gel permeation chromatography (GPC) confirming successful CPP synthesis. c) UV–vis absorption spectra of CPP LNPs at varying molar ratios (5%, 10%, and 30%). d) Size distribution of CPP LNPs (*n* = 3). e) Encapsulation efficiency (%) of negative control siRNA in CPP LNPs with varying CPP content, as determined by the RiboGreen assay. f) Western blot analysis of CPP LNP‐mediated siRNA transfection efficiency at different molar ratios (5%, 10%, and 30%). g) Comparison of SOSG fluorescence intensity in PBS, LNP, and 5% or 10% CPP LNPs after laser irradiation. h) Cryo‐TEM images of siGPX4 LNPs (left) and siGPX4 PLNPs (right), demonstrating spherical morphology and uniform particle size distribution. Data are presented as the mean ± SD (*n* = 3). Statistical significance was determined as follows: ns, not significant; ^**^ *p* <0.01; ^****^ *p* <0.0001.

In this study, we synthesized a photosensitizing lipid, cholesterol–PEG–pheophorbide a (CPP), to fabricate photosensitizing lipid nanoparticles (PLNPs). CPP was synthesized via an amide coupling reaction between the carboxylic acid group of pheophorbide a (pheo a) and the amine terminus of cholesterol‐PEG‐amine. The reaction was facilitated by N,N′‐diisopropylcarbodiimide (DIC) and N‐hydroxysuccinimide (NHS) as coupling agents. Following the reaction, the crude product was purified by silica gel chromatography. The structure of the synthesized CPP was confirmed by proton nuclear magnetic resonance (^1^H NMR) spectroscopy.^[^ ^31^ ^]^ Characteristic aromatic peaks of pheo a appeared in the 9.9–7.9 ppm range, while the PEG signals were observed ≈3.6 ppm. Additionally, the characteristic signals of cholesterol were detected in the aliphatic region between 1.3 and 0.6 ppm (**Figure** **1**a; Figure S1, Supporting Information). Gel permeation chromatography (GPC) analysis revealed a decrease in retention time compared with the starting materials, indicating an increase in molecular size due to successful conjugation (Figure 1b).^[^ ^32^, ^33^ ^]^ The molecular weight of the CPP was further confirmed by LC–TOF–MS analysis (Figure S2, Supporting Information). The mass spectrum displayed a series of peaks separated by 22 Da in m/z, corresponding to PEG repeat units (44 Da) observed in the doubly charged state (z = 2). The dominant peak at m/z 1255.3 corresponded to a molecular mass of ≈2509 Da, which was in good agreement with the theoretical mass of the CPP conjugate. Collectively, these results verified the successful formation of an amide bond between cholesterol–PEG–amine and pheo a and confirmed the expected oligomeric distribution of PEG. Moreover, CPP demonstrated good photostability under laser irradiation, as indicated by unchanged UV–Vis absorption spectra before and after irradiation (Figure S3, Supporting Information).

To evaluate the physicochemical properties of the nanoparticles, CPP LNPs were formulated using the synthesized CPP and a negative control siRNA (siNC).^[^ ^34^ ^]^ Based on the typical molar ratio of cholesterol in conventional LNPs (38.5%), CPP was incorporated at 5%, 10%, and 30% substitution levels, corresponding to 1.93%, 3.85%, and 11.55% of the total lipid content, respectively. In total, four different LNP formulations—including a control LNP without CPP—were prepared for comparison (Table S1, Supporting Information). To assess the optical properties of the CPP LNPs, UV–vis absorption spectroscopy was performed. CPP exhibited a characteristic absorption peak near 670 nm, which was consistent with the known spectral properties of pheo a (Figure S4, Supporting Information).^[^ ^35^ ^]^ Moreover, the absorbance intensity increased with higher CPP content, confirming successful encapsulation of pheo a in the CPP LNPs (Figure 1c). Dynamic light scattering (DLS) analysis was conducted to assess particle size and distribution. The 5% CPP LNPs (106.6 nm) and 10% CPP LNPs (110.2 nm) exhibited particle sizes and polydispersity index (PDI) values (≈0.2–0.3) similar to the control LNPs, indicating the formation of small, uniform nanoparticles. However, the 30% CPP LNPs exhibited a marked increase in particle size and polydispersity (Figure 1d).^[^ ^36^ ^]^ The siRNA encapsulation efficiency of CPP LNPs was also evaluated. Both the 5% and 10% CPP LNPs showed high encapsulation efficiencies exceeding 80%, comparable to the control LNP. In contrast, the 30% CPP LNPs showed a significant reduction in encapsulation efficiency, dropping below 50% (Figure 1e).^[^ ^34^ ^]^ To further optimize the lipid composition of the CPP‐based LNPs, a modified formulation (10% CPP LNP_1) was prepared by replacing 1,2‐dimyristoyl‐rac‐glycero‐3‐methoxypolyethylene glycol‐2000 (DMG‐PEG2k) with CPP at an equivalent molar ratio, based on the original 10% CPP LNP formulation (Table S2, Supporting Information). DLS analysis revealed that the 10% CPP LNP maintained a stable size (147.5 nm) and low PDI (0.023), while the 10% CPP LNP_1 exhibited a dramatically increased particle size (9087 nm) and a PDI of 1.0, indicating failure to form nanoscale particles (Figure S5, Supporting Information). These results suggest that DMG‐PEG2k is essential for proper LNP formation and stability, and that CPP content should be modulated by adjusting the cholesterol proportion, not by replacing DMG‐PEG2k. To evaluate transfection efficiency, LNPs encapsulating GAPDH siRNA were delivered to 4T1 cells. A marked decrease in gene silencing efficiency was observed for the 30% CPP LNP formulation (Figure 1f). Based on these findings, the 30% CPP LNP was excluded from further experiments, and optimization focused on the remaining formulations. To identify the optimal PLNP composition, various formulations encapsulating siNC were evaluated for PDT efficacy, with ROS generation serving as an indicator. Measurement of singlet oxygen sensor green (SOSG) fluorescence intensity showed that the 10% CPP LNP generated ROS more effectively than the 5% CPP LNP in a laser intensity‐dependent manner (Figure 1g). These results indicate that the 10% CPP LNP formulation is more effective for PDT. Intracellular ROS generation was further assessed in 4T1 cells using the fluorescent probe DCFH‐DA.^[^ ^37^ ^]^ Cells treated with the 10% CPP LNP produced significantly higher ROS levels than those treated with the 5% CPP LNP (Figure S6a, Supporting Information). Lipid peroxidation was also evaluated using the ‐BODIPY 581/591 C11 probe following laser irradiation.^[^ ^27^ ^]^ The 10% CPP LNP treatment group showed significantly greater lipid peroxidation accumulation compared with the 5% CPP LNP group (Figure S6b, Supporting Information), indicating enhanced induction of lipid peroxidation. These results demonstrate that the 10% CPP LNP formulation provides superior PDT efficacy compared with the 5% CPP LNPs in 4T1 cells, owing to stronger induction of ROS production and lipid peroxidation. Given its superior performance, the 10% CPP LNP was selected as the optimized PLNP formulation for GPX4‐targeting siRNA (siGPX4) encapsulation. To confirm its structural integrity, we next prepared conventional LNPs and siGPX4‐loaded PLNPs for Cryogenic Transmission Electron Microscopy (Cryo‐TEM) analysis (Figure 1h). Cryo‐TEM imaging revealed that both formulations exhibited uniform, spherical morphology consistent with DLS data. The 10% CPP LNP maintained a dense core‐shell structure without multilamellar features, indicating that CPP incorporation does not disrupt the intrinsic non‐lamellar architecture of MC3‐based LNPs. Finally, storage stability was assessed by monitoring the z‐average size, PDI, and zeta potential over 14 days, revealing minimal changes and confirming high colloidal stability (Figure S7, Supporting Information).

### 2.2 Evaluation of In Vitro Gene Delivery Efficiency and GPX4 Gene Silencing Effect of PLNPs

**Figure 2:**
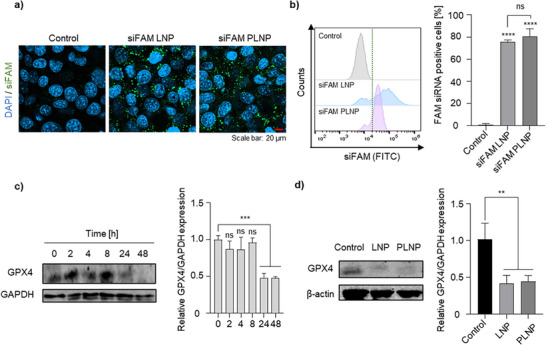
In vitro assessment of gene delivery efficiency and GPX4 gene silencing by PLNPs. a) Representative CLSM images showing cellular uptake of siFAM (green). b) Flow cytometry histograms depicting intracellular distribution of siFAM. c) Representative images of GPX4 protein expression in 4T1 cells over time following treatment with siGPX4‐loaded PLNPs. Quantitative densitometric analysis (mean ± SD, *n* = 3) is shown alongside the blots. d) Representative western blot analysis of GPX4 protein levels in 4T1 cells after treatment with LNPs or PLNPs. Data are presented as the mean ± SD (*n* = 3). Statistical significance was determined as follows: ns, not significant; ^*^ *p* <0.05; ^**^ *p* <0.01; ^***^ *p* <0.001; ^****^ *p* <0.0001.

To evaluate the gene delivery capability of the PLNPs, cellular internalization of siRNA was initially assessed using fluorescein‐labeled negative control siRNA (siFAM). 4T1 cells were incubated with siFAM‐loaded LNPs (siFAM LNP) or PLNPs (siFAM PLNP), and intracellular fluorescence was visualized using confocal laser scanning microscopy (CLSM). Compared with untreated control, both treatment groups exhibited strong intracellular green fluorescence, indicating successful siRNA uptake (**Figure** **2**a).^[^ ^38^, ^39^, ^40^ ^]^ These observations were further confirmed by quantitative flow cytometry analysis, which showed that the siFAM PLNP group displayed a fluorescence intensity comparable to that of the siFAM LNP group, suggesting that PLNPs achieve siRNA delivery efficiency similar to conventional LNPs (Figure 2b). After confirming efficient siRNA delivery, the intracellular delivery of siGPX4 PLNPs in 4T1 breast cancer cells was evaluated. The encapsulation efficiency of siGPX4 in PLNPs was first determined, showing a value of 86.85%, which was comparable to that of siNC‐loaded PLNPs, indicating stable siRNA incorporation. The gene silencing effect of siGPX4 was then examined. RT‐PCR analysis revealed that GPX4 mRNA expression began to decrease 2 h after treatment, was almost completely suppressed at 4 h, and partially recovered by 24 h (Figure S8, Supporting Information). In contrast, GPX4 protein levels exhibited a delayed response, showing a marked reduction at 24 h post‐treatment (Figure 2c), reflecting the expected lag between transcriptional and translational regulation following siRNA‐mediated silencing. Western blot analysis performed 24 h after treatment with siGPX4 LNPs and siGPX4 PLNPs further confirmed efficient GPX4 knockdown in both formulations (Figure 2d). Based on these findings, laser irradiation was performed 24 h post‐treatment, when siGPX4 knockdown was maximal. This timing ensured sufficient suppression of the antioxidant defense system, allowing photodynamically generated ROS to induce maximal synergistic effects, whereas earlier time points (6–12 h) showed negligible knockdown. Collectively, these results demonstrate that PLNPs can efficiently deliver GPX4 siRNA, achieve potent gene silencing, and potentially promote ferroptosis. Subsequent experiments were therefore conducted to investigate ferroptosis induction and the synergistic effect of combining GPX4 silencing with PDT using siGPX4 PLNPs.

### 2.3 Synergistic Effect of Ferroptosis Induction via GPX4 Inhibition and PDT

**Figure 3:**
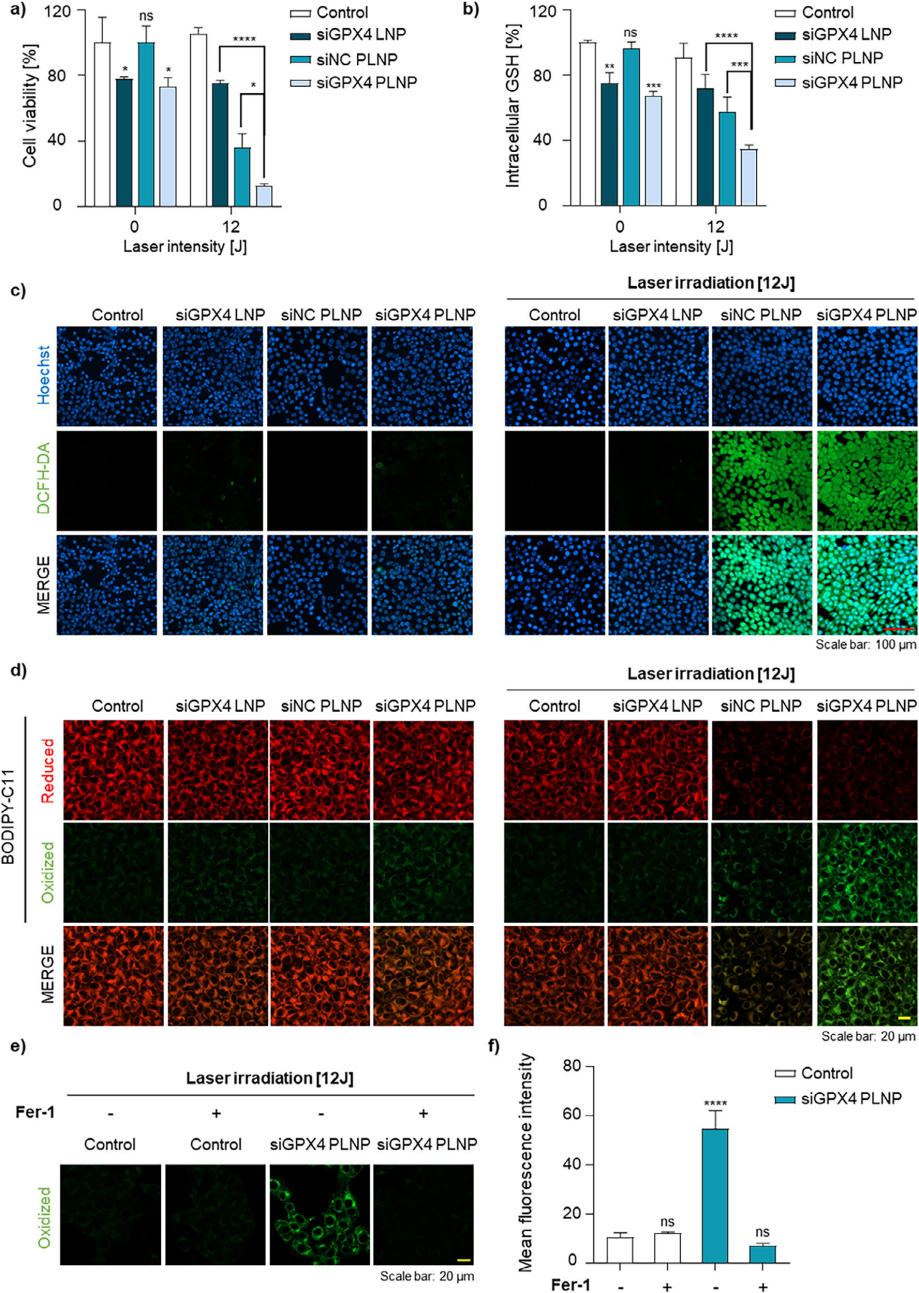
Synergistic induction of ferroptosis by GPX4 inhibition combined with PDT. a) Cell viability of 4T1 cells treated with siGPX4 LNP, siNC PLNP, or siGPX4 PLNP, under or without laser irradiation. b) Intracellular GSH levels in 4T1 cells after treatment with the above formulations, under or without laser irradiation. c) Representative CLSM images of 4T1 cells stained with DCFH‐DA to detect ROS, after treatment with siGPX4 LNP, siNC PLNP, or siGPX4 PLNP, with or without laser irradiation. Scale bars: 100 µm. d) Representative CLSM images of 4T1 cells stained with BODIPY‐C11 to assess lipid peroxidation, after treatment with siGPX4 LNP, siNC PLNP, or siGPX4 PLNP, under or without laser irradiation. Scale bars: 20 µm. e) CLSM images of 4T1 cells treated with siGPX4 PLNP and ferrostatin‐1 (Fer‐1), followed by laser irradiation and staining with BODIPY‐C11. Scale bars: 20 µm. f) Quantitative analysis of BODIPY‐C11 fluorescence intensity from panel (e). Data are presented as the mean ± SD (*n* = 3). Statistical significance was determined as follows: ns, not significant; ^*^ *p* <0.05; ^**^ *p* <0.01; ^***^ *p* <0.001; ^****^ *p* <0.0001.

This study aimed to induce ferroptosis through PDT‐mediated oxidative stress by effectively delivering GPX4 siRNA using PLNPs and to evaluate the resultant antitumor efficacy. All subsequent experiments were conducted with laser irradiation performed 24 h after LNP treatment, based on the earlier results. Initially, western blot analysis was conducted 24 h after treatment with siGPX4 LNPs or siGPX4 PLNPs, followed by laser irradiation. The results showed a decreased GPX4 protein expression in all experimental groups (Figure S9, Supporting Information). To assess the synergistic effect of GPX4 silencing and PDT, 4T1 cells were treated with PBS (control), siGPX4 LNP, siNC PLNP, or siGPX4 PLNP, followed by laser irradiation at 24 h. Cell viability was then measured using the CCK assay. The siGPX4 LNP group exhibited slight cytotoxicity regardless of irradiation, attributed to GPX4 inhibition. In the siNC PLNP group, laser treatment reduced cell viability to ≈40%, reflecting PDT‐induced oxidative stress. Notably, the siGPX4 PLNP group showed minimal toxicity without laser exposure but demonstrated near‐complete cell death after irradiation (**Figure** **3**a).

Intracellular glutathione (GSH) levels were subsequently analyzed under the same treatment conditions. In the siGPX4 LNP group, GSH levels modestly declined because of GPX4 inhibition, independent of laser treatment. In contrast, GSH depletion in the siNC PLNP group was observed only after irradiation, attributed to ROS generation via PDT.^[^ ^41^, ^42^ ^]^ The most substantial GSH reduction occurred in the siGPX4 PLNP group upon laser treatment, demonstrating a synergistic effect of GPX4 inhibition and PDT‐induced oxidative stress. These findings suggest that siGPX4 PLNP effectively induces ROS production, leading to GSH depletion (Figure 3b).^[^ ^42^, ^43^, ^44^ ^]^ To assess ROS production, DCFH‐DA fluorescence staining was performed. The strongest fluorescence signal was observed in the siGPX4 PLNP group following laser irradiation, indicating elevated ROS generation (Figure 3c). Lipid peroxidation, evaluated via BODIPY‐C11 staining, showed the most pronounced decrease in red fluorescence (reduced form) and an increase in green fluorescence (oxidized form) in the siGPX4 PLNP‐treated group, further supporting enhanced lipid peroxidation (Figure 3d). Ferroptosis is characterized by distinct mitochondrial damage, including reduced cristae, membrane condensation, and ruptured outer membranes of deformed mitochondria.^[^ ^45^ ^]^ To assess mitochondrial membrane potential, JC‐1 staining was employed. JC‐1 aggregates fluoresce red in healthy mitochondria, whereas green fluorescence indicates depolarized membranes.^[^ ^46^, ^47^ ^]^ JC‐1 staining revealed increased green fluorescence in the siGPX4 PLNP group under laser irradiation, suggesting mitochondrial dysfunction consistent with ferroptosis (Figure S10, Supporting Information).^[^ ^48^, ^49^ ^]^ To confirm that the therapeutic effects of GPX4 silencing and PDT were mediated via the ferroptosis pathway, cells were pretreated with the ferroptosis inhibitor, ferrostatin‐1 (Fer‐1).^[^ ^50^ ^]^ Fer‐1 suppressed the laser‐induced increase in oxidized fluorescence in siGPX4 PLNP‐treated cells (Figure 3e), and reduced lipid ROS levels to near‐control levels (Figure 3f),^[^ ^27^ ^]^ validating that cell death was mediated by ferroptosis.^[^ ^51^ ^]^ Finally, the photodynamic therapeutic efficacy of siGPX4 PLNPs was validated in another breast cancer model using EO771 cells. Similar trends in reduced viability, GSH depletion, elevated ROS generation, and lipid peroxidation were observed following siGPX4 PLNP treatment and irradiation, consistent with results from the 4T1 cell line (Figure S11, Supporting Information). Overall, these results suggest that the combined inhibition of GPX4 and application of PDT significantly enhances anticancer effects by inducing ferroptosis. siGPX4 PLNPs compromise the intracellular antioxidant system via GPX4 suppression, amplify ROS production under light exposure, and intensify lipid peroxidation, thereby presenting a novel and potent therapeutic strategy that addresses limitations of conventional PDT.

### 2.4 In Vivo Intratumoral Retention and Therapeutic Efficacy of siGPX4 PLNP

**Figure 4:**
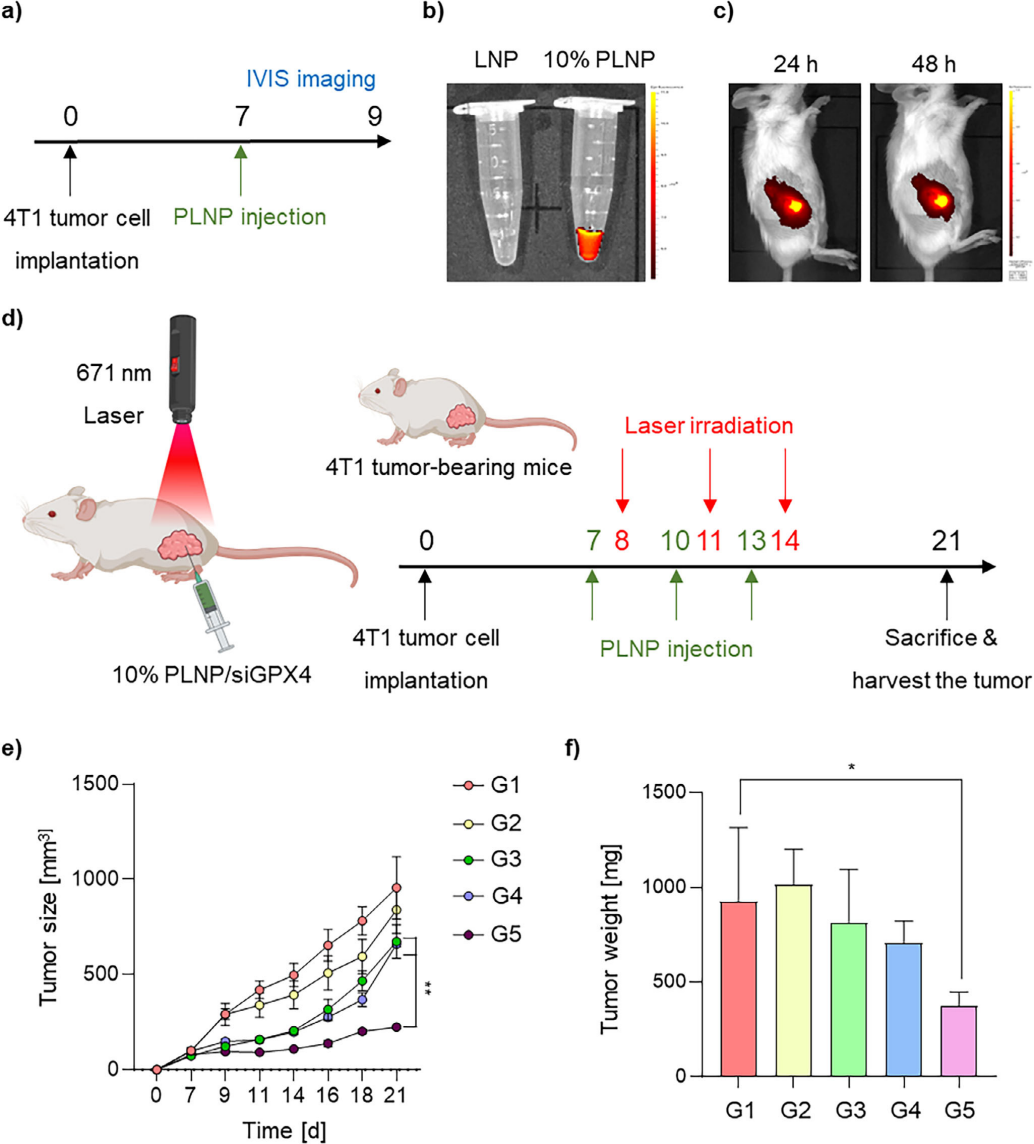
In vivo intratumoral retention and therapeutic efficacy of siGPX4 PLNP. a) Schematic of the experimental protocol using 4T1 tumor‐bearing mice. b) Fluorescence image (pheo a) of Eppendorf tubes containing LNP (left) and 10% PLNP (right). c) Time‐dependent in vivo fluorescence imaging of tumors at 24 and 48 h post‐injection. d) Schematic overview of the in vivo therapeutic protocol using siGPX4 PLNP. e) Tumor growth curves of mice treated with PBS, siNC PLNP, or siGPX4 PLNP, with or without laser irradiation. f) Mean tumor weights in each group. Data are presented as the mean ± SD (*n* = 4). Statistical significance was determined as follows: ^*^ *p* <0.05; ^**^ *p* <0.01. Group 1 (G1): PBS (control); Group 2 (G2): siNC PLNP; Group 3 (G3): siGPX4 PLNP; Group 4 (G4): siNC PLNP + Laser; Group 5 (G5): siGPX4 PLNP + Laser.

The retention of PLNPs within tumors was evaluated using an in vivo imaging system (IVIS) at 24 and 48 h post‐administration (**Figure** **4**a).^[^ ^52^ ^]^ Prior to in vivo imaging, the intrinsic pheo a fluorescence intensity of PLNPs was measured and found to be significantly higher than that of control LNPs (Figure 4b). Following direct intratumoral injection, PLNPs demonstrated stable retention within the tumor for up to 48 h (Figure 4c). Ex vivo fluorescence imaging of major organs harvested 48 h after administration confirmed selective retention of PLNPs in tumor tissue (Figure S12, Supporting Information). To assess the antitumor efficacy of siGPX4‐loaded PLNPs combined with PDT, a breast cancer mouse model was used. Mice were intratumorally injected with PBS (control), siNC PLNPs, or siGPX4 PLNPs (Figure 4d). Laser irradiation (671 nm, 100 J) was applied 24 h post‐injection, with a total of three nanoparticle injections and three subsequent irradiation sessions—each performed 1 d after nanoparticle administration. Tumor growth was monitored for 7 d after the final laser irradiation. A significant inhibition of tumor growth was observed only in the group treated with siGPX4 PLNPs followed by laser irradiation (Figures 4e,f; S13, Supporting Information). This outcome is particularly meaningful considering that the 4T1 breast tumor model is highly aggressive and metastatic, making therapeutic suppression extremely challenging.^[^ ^53^, ^54^, ^55^ ^]^ Importantly, the therapeutic effect was supported by mechanistic evidence, including GPX4 knockdown and enhanced lipid peroxidation, which confirms the therapeutic relevance of our strategy.^[^ ^56^ ^]^ Collectively, these findings demonstrate that PLNPs exhibit prolonged retention within the tumor microenvironment, thereby enhancing gene delivery efficiency and photodynamic efficacy to promote ferroptosis and suppress tumor progression.

### 2.5 In Vivo Analysis of Ferroptosis Enhancement via GPX4 Knockdown and PDT

**Figure 5:**
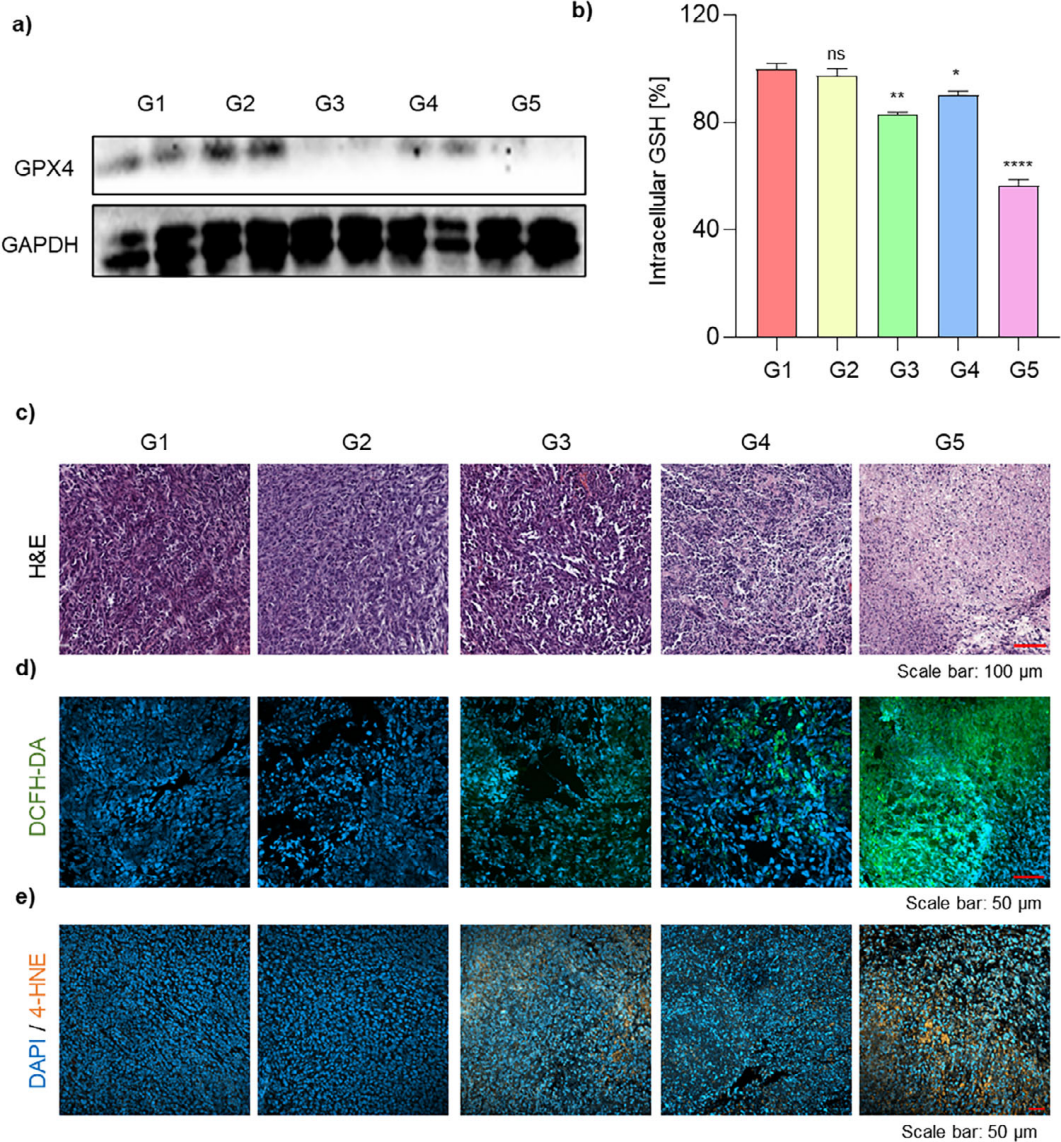
In vivo evaluation of ferroptosis enhancement via GPX4 knockdown combined with PDT. a) Western blot analysis of GPX4 and GAPDH protein levels in tumor lysates from each treatment group. b) Relative GSH levels in tumor tissues. Data are presented as the mean ± SD (*n* = 4). Statistical significance was determined as follows: ns, not significant; ^*^ *p* <0.05; ^**^ *p* <0.01; ^****^ *p* <0.0001. c) H&E staining of tumor sections showing histopathological changes after each treatment. d) CLSM images of tumor sections stained with DCFH‐DA for ROS detection. e) Immunofluorescence staining of tumor sections for 4‐HNE to evaluate lipid peroxidation. Group 1 (G1): PBS (control); Group 2 (G2): siNC PLNP; Group 3 (G3): siGPX4 PLNP; Group 4 (G4): siNC PLNP + Laser; Group 5 (G5): siGPX4 PLNP + Laser.

The enhancement of ferroptosis through GPX4 gene knockdown combined with PDT was evaluated. Western blot analysis of tumor lysates revealed a substantial reduction in GPX4 protein expression in groups treated with GPX4 siRNA (G3 and G5) compared with control groups (G1, G2, and G4) (**Figure** **5**a). Immunofluorescence staining of tumor sections further confirmed significantly reduced GPX4 fluorescence signals exclusively in the G3 and G5 groups (Figure S14, Supporting Information), indicating success in vivo gene delivery via the PLNP formulation. In alignment with reduced GPX4 expression, intracellular GSH levels—critical for regulating ferroptosis—were significantly lower in tumor tissues from the siRNA‐treated groups (G3 and G5), compared with controls (Figure 5b). The most pronounced GSH depletion was observed in the siGPX4 PLNP group following laser irradiation (G5), suggesting a synergistic induction of ferroptosis through combined GPX4 inhibition and PDT. Given that GPX4 utilizes GSH as a cofactor to exert its antioxidant activity, their simultaneous depletion indicates a collapse of the cellular antioxidant defense system, thereby creating a pro‐ferroptotic environment.^[^ ^57^, ^58^ ^]^ Histopathological analysis of tumor sections using H&E staining (Figure 5c) revealed the most significant morphological changes and extensive cell death in the G5 group, demonstrating a potent antitumor effect resulting from the combination of siGPX4 PLNPs and PDT.^[^ ^59^ ^]^ To further elucidate the mechanism of cell death, ROS generation and lipid peroxidation were assessed. Considering the transient nature of ROS, tumor sections were collected 24 h after the final laser irradiation for ROS analysis.^[^ ^60^ ^]^ CLSM imaging of DCFH‐DA‐stained tumor sections revealed that ROS levels in the GPX4 knockdown (G3) and PDT‐only (G4) groups were relatively lower compared with those in the combined treatment group (G5). In contrast, the G5 group exhibited the highest ROS levels (Figure 5d), indicating a synergistic enhancement of oxidative stress due to GSH depletion and GPX4 suppression. Additionally, immunofluorescence staining for 4‐hydroxynonenal (4‐HNE), a key marker of lipid peroxidation, revealed significantly increased 4‐HNE levels in the G5 group (Figure 5e).^[^ ^6^ ^]^ These findings confirm effective induction of lipid peroxidation, a defining characteristic of ferroptosis. To assess the biocompatibility of PLNPs, body weight monitoring, blood biochemical analysis, and histological examination were performed (Figures S15–S17, Supporting Information). No significant changes in body weight were observed across treatment groups. Blood chemistry and histopathological analyses also confirmed the excellent biocompatibility of PLNPs, with no observable adverse effects.^[^ ^61^ ^]^ Collectively, these in vivo findings demonstrate that the combination of GPX4 knockdown via siRNA and PDT effectively induces ferroptosis in tumor tissues.^[^ ^28^, ^29^ ^]^ This was evidenced by reduced GPX4 expression, GSH depletion, elevated ROS production, increased lipid peroxidation (4‐HNE), and extensive tumor cell death confirmed through histological analysis. The combinatorial therapeutic strategy proposed in this study presents a promising approach for ferroptosis‐based targeted cancer therapy and may offer a new paradigm for future anticancer treatments.

## 3 Conclusion 

In this study, we present an innovative tumor treatment strategy that effectively induces ferroptosis in vivo through the combined use of PLNPs for GPX4 siRNA delivery and PDT. The engineered PLNPs successfully delivered siRNA to suppress GPX4 gene expression. This suppression, coupled with GSH depletion and PDT‐induced ROS generation, produced a synergistic effect that maximized intracellular ROS accumulation.^[^ ^62^, ^63^ ^]^ Our in vivo experiments demonstrated that this synergistic interaction significantly inhibited tumor growth while causing minimal systemic toxicity, highlighting both the therapeutic potential and safety of the proposed platform. This approach addresses several key limitations of conventional PDT, including poor photosensitizer solubility, limited tissue penetration, systemic toxicity, and inadequate tumor specificity.^[^ ^64^ ^]^ The LNP‐based formulation contributed to improved therapeutic efficacy by enhancing photosensitizer solubility, protecting siRNA, and utilizing the intrinsic biocompatibility and biodegradability of LNPs. Moreover, the favorable safety profile—confirmed through systemic toxicity assessments and histopathological analysis of major organs—strongly supports the clinical translational potential of siGPX4 PLNPs. However, as this study employed intratumoral injections for therapeutic evaluation, further investigations are necessary to optimize systemic delivery methods (e.g., intravenous administration) and tumor‐targeting strategies for clinical application. To this end, our research group is currently exploring various ligand‐based targeting approaches to facilitate selective tumor accumulation of PLNPs following intravenous administration. Collectively, our findings suggest that siGPX4 PLNPs represent a promising therapeutic platform for enhancing the clinical efficacy of PDT. Future studies involving diverse tumor models and the optimization of treatment parameters will be essential to validate the safety and efficacy of this strategy and support its development as a more effective and clinically viable therapeutic option.

## 4 Experimental Section

### Materials

DLin‐MC3‐DMA (MC3) was purchased from MedChemExpress (Monmouth Junction, NJ, USA). 1,2‐Distearoyl‐sn‐glycero‐3‐phosphocholine (DSPC) and 1,2‐dimyristoyl‐rac‐glycero‐3‐methoxypolyethylene glycol‐2000 (DMG‐PEG2k) were obtained from Avanti Polar Lipids (Alabaster, AL, USA). Cholesterol, ethyl alcohol, N‐hydroxysuccinimide (NHS), and N,N′‐diisopropylcarbodiimide (DIC) were purchased from Sigma–Aldrich (St. Louis, MO, USA). Pheophorbide a was obtained from Frontier Scientific (Newark, DE, USA). Dichloromethane and methyl alcohol were acquired from Daejung Chemicals (Gyeonggi, Republic of Korea). Cholesterol‐PEG‐amine (molecular weight: 2 k) was procured from Biopharma PEG (Watertown, MA, USA). Negative control siRNA and fluorescein‐labeled siNC (siFAM) were purchased from Bioneer (Daejeon, Korea).

### Synthesis and Characterization of Cholesterol–PEG–Pheophorbide a (CPP)

Cholesterol–PEG–Pheophorbide a (CPP) was synthesized via DIC/NHS‐mediated amide coupling between pheophorbide a and cholesterol–PEG–amine. First, pheophorbide a (24 mg, 1 equiv.) was dissolved in dichloromethane (DCM, 400 µL). DIC (6.05 mg, 1.2 equiv.) and NHS (6.90 mg, 1.5 equiv.) were added to the solution, and the reaction was stirred at room temperature for 4 h to activate the carboxyl group. Separately, cholesterol‐PEG‐amine (80 mg, 1 equiv.) was dissolved in DCM (400 µL) containing triethylamine (TEA, 8.1 mg, 2 equiv.) and stirred for 15 min. The activated pheophorbide a solution was then added dropwise to the cholesterol‐PEG‐amine solution, and the combined mixture was stirred at room temperature for 24 h. Reaction progress was monitored via thin‐layer chromatography (TLC). After 24 h, the mixture was concentrated under reduced pressure, and the crude residue was purified by silica gel column chromatography using a DCM/methanol (12.5:1 v/v) eluent. CPP was obtained as a dark green solid with a yield of 68.6% after purification. The purified CPP product was obtained as a solid. Chemical structures and reaction schemes were drawn using MarvinSketch, Chemaxon (https://www.chemaxon.com).

### Gel Permeation Chromatography (GPC) Analysis

Gel permeation chromatography (GPC) was carried out on a PLgel mixed column using tetrahydrofuran (THF) as the eluent at a flow rate of 1.0 mL min^−1^. The column temperature was maintained at 40 °C. Samples were prepared at a concentration of 1.0 mg mL^−1^ and injected with a volume of 50 µL. Calibration was performed using polystyrene standards.

### Preparation of Cholesterol–PEG–Pheophorbide a Lipid Nanoparticles (CPP LNPs)

CPP LNPs were formulated by dissolving MC3, DSPC, cholesterol, and DMG‐PEG2k in ethanol at a molar ratio of 50:10:38.5:1.5. To obtain CPP‐incorporated formulations, cholesterol was partially substituted with CPP while maintaining the combined molar fraction of cholesterol + CPP at 38.5% (Table S1, Supporting Information). This lipid solution was mixed with siRNA in 10 mm citrate buffer (pH 4) at a volume ratio of 1:3, maintaining an N/P ratio of 4. Rapid mixing was achieved by vigorous vortexing or by microfluidic assembly (NanoKRAShoT; KRON, South Korea), after which the nanoparticles were dialyzed twice against 1X PBS (pH 7.4) using a 10 kDa molecular weight cutoff (MWCO) centrifugal filter (3,000 g, 20 min each), and stored at 4 °C.

### Characterization of CPP LNPs

CPP LNPs were characterized using UV–vis spectrophotometry (UV‐1280; Shimadzu, Japan) and dynamic light scattering (DLS; Malvern Nano ZS, Malvern Instruments Ltd., UK). The encapsulation efficiency of siRNA was measured using the RiboGreen RNA assay kit (Thermo Fisher Scientific, MA, USA) according to the manufacturer's protocol. Briefly, LNPs were lysed with Triton X‐100 to release encapsulated siRNA, which was then stained with the RiboGreen reagent. Fluorescence intensity was measured at excitation/emission wavelengths of 485/530 nm using a microplate reader (Synergy H1; BioTek, Winooski, VT, USA). The encapsulated RNA (internal siRNA) was calculated by subtracting the external (unencapsulated) siRNA from the total siRNA. Encapsulation efficiency (%) was defined as the proportion of internal siRNA relative to the total siRNA.^[^ ^34^ ^]^ The morphological characteristics of the LNPs were further examined by cryogenic transmission electron microscopy (Glacios; Thermo Fisher Scientific, MA, USA).

### In Vitro Cell Experiment

Mouse breast cancer cell lines 4T1 and EO771 were obtained from the American Type Culture Collection (ATCC; Manassas, VA, USA). Cells were cultured in either Roswell Park Memorial Institute (RPMI) 1640 medium or Dulbecco's Modified Eagle Medium (DMEM), supplemented with 10% fetal bovine serum (FBS) and 1% antibiotic‐antimycotic (A/A) solution. All cells were maintained at 37 °C in a humidified incubator with 5% CO_2_.

### Measurement of Singlet Oxygen Generation

Singlet oxygen production was assessed using the Singlet Oxygen Sensor Green (SOSG; Invitrogen, USA) reagent. A stock solution of SOSG in methanol was diluted with distilled water to obtain a 2 µm working solution. Then, 100 µL of the SOSG solution was mixed 1:1 with 100 µL of each sample. The samples were irradiated using a 671 nm laser (100 mW). Fluorescence intensity (excitation/emission: 494/534 nm) was measured using a microplate reader (Synergy H1; BioTek, Winooski, VT, USA).

### Cellular Uptake Analysis

For visualization of cellular internalization, 4T1 cells (1 × 10⁵ cells mL^−1^) were seeded in confocal dishes and incubated with siFAM LNP or siFAM PLNP containing 500 ng of FAM‐labeled siRNA per well for 24 h at 37 °C. After washing with PBS, cellular uptake was visualized using a confocal laser scanning microscope (LSM 700; Carl Zeiss). For flow cytometry analysis, 4T1 cells (1 × 10⁵ cells mL^−1^) were seeded in 12‐well plates and incubated with siFAM LNP or siFAM PLNP containing 500 ng of FAM‐labeled siRNA per well for 24 h. The cells were harvested and analyzed using a MA900 Multi‐Application Cell Sorter (Sony Biotechnology, San Jose, CA, USA) (NFEC‐2024‐10‐300262).

### Cytotoxicity Assessment

4T1 cells (1 × 10⁵ cells mL^−1^) were seeded in 96‐well plates and incubated with siGPX4 LNP or siGPX4 PLNP containing 100 ng of siGPX4 per well for 24 h at 37 °C. Cells were then either irradiated with a 671 nm laser (100 mW, 120 s) or left untreated as controls, followed by an additional 24 h incubation. Cell viability was determined using the Cell Counting Kit‐8 (CCK‐8) assay after 1 h incubation with the working solution. Absorbance was measured at 450 nm using a microplate reader (Synergy H1; BioTek, Winooski, VT, USA).

### Intracellular GSH Level Detection

4T1 cells (1 × 10⁵ cells mL^−1^) were seeded in 6‐well plates and treated with siGPX4 LNP or siGPX4 PLNP containing 2 µg of siGPX4 per well for 24 h at 37 °C. Following treatment, cells were harvested and lysed using three freeze–thaw cycles (10 min in liquid nitrogen followed by 10 min at 37 °C). The lysates were centrifuged at 14,000 rpm for 10 min at 4 °C. The resulting supernatants were analyzed immediately using a GSH assay kit (Invitrogen). Absorbance was measured at 405 nm using a microplate reader (Synergy H1; BioTek, Winooski, VT, USA).

### Intracellular ROS Detection

Intracellular ROS levels were measured using the fluorescent probe 2′,7′‐dichlorofluorescein diacetate (DCFH‐DA). Briefly, 4T1 cells (1 × 10⁵ cells mL^−1^) were seeded into confocal dishes and incubated with siGPX4 LNP, siNC PLNP, or siGPX4 PLNP containing 500 ng of siGPX4 per well for 24 h at 37 °C. After washing with PBS, the cells were stained with DCFH‐DA (30 µm) for 30 min at 37 °C and subsequently irradiated with a 671 nm laser (100 mW, 120 s). Hoechst (Invitrogen) was used for nuclear staining. Following another PBS wash, ROS generation was visualized using confocal laser scanning microscopy (CLSM; LSM 700; Carl Zeiss, Germany).

### In Vitro Lipid Peroxidation Detection

Lipid peroxidation was assessed using BODIPY 581/591 C11, a fluorescent probe that emits green fluorescence upon oxidation by lipid peroxides. 4T1 cells (1 × 10⁵ cells mL^−1^) were seeded into confocal dishes and incubated with siGPX4 LNP, siNC PLNP, or siGPX4 PLNP containing 500 ng of siGPX4 per well for 24 h at 37 °C. After incubation, cells were either irradiated with a 671 nm laser (100 mW, 120 s) or left untreated as controls. Cells were then stained with BODIPY‐C11 (2 µm) for 30 min at 37 °C. The labeled cells were imaged using confocal laser scanning microscopy (CLSM). Fluorescence was monitored at excitation/emission wavelengths of 495/519 nm for oxidized BODIPY‐C11 and 596/615 nm for the reduced form.

### Western Blot Assay

Cells were lysed using PRO‐PREP lysis buffer (iNtRON Biotechnology, Seongnam, South Korea) supplemented with a 1X phosphatase inhibitor cocktail (Roche Applied Science, Indianapolis, IN, USA). Protein concentrations were determined using a BCA assay kit (Thermo Fisher Scientific). Equal amounts of protein were separated by SDS‐PAGE and transferred onto nitrocellulose membranes. Membranes were blocked with 5% bovine serum albumin (BSA), followed by overnight incubation at 4 °C with specific primary antibodies. After washing, membranes were incubated for 1 h at room temperature with horseradish peroxidase‐conjugated secondary antibodies. Protein bands were visualized using enhanced chemiluminescence reagents (SuperSignal West Pico PLUS; Thermo Fisher Scientific). GAPDH and β‐actin were used as a loading control.

### RT‐PCR Analysis

Total RNA was extracted at different time points (0, 2, 4, 8, 24, and 48 h) after siGPX4 PLNP treatment, and cDNA was synthesized using HiSense cDNA Synthesis Master Mix (Cellsafe). GPX4 mRNA levels were quantified by RT‐PCR with specific primers (listed in Table S4, Supporting Information). Relative expressions were normalized to RPL7 as an internal control.

### Evaluation of Ex Vivo Fluorescence Imaging

To evaluate the retention of PLNP in tumor tissue, ex vivo fluorescence imaging was performed at 24 and 48 h after intratumoral injection in a 4T1 tumor model using the IVIS Lumina XRMS In Vivo Imaging System (PerkinElmer, USA) located at the BIORP of the Korea Basic Science Institute (KBSI). At 48 h post‐injection, tumors and major organs were harvested, and organ‐specific fluorescence imaging was conducted.

### In Vivo Antitumor Efficacy

Seven‐week‐old BALB/c mice were inoculated subcutaneously with 4T1 cells (1 × 10⁶ cells mouse^−1^) in the right flank. When tumors reached ≈100 mm^3^ (day 7 post‐inoculation), mice were randomly divided into five groups (*n* = 6 per group): Group 1 (G1): PBS (control); Group 2 (G2): siNC PLNP; Group 3 (G3): siGPX4 PLNP; Group 4 (G4): siNC PLNP + Laser; and Group 5 (G5): siGPX4 PLNP + Laser. Lipid nanoparticles (siRNA: 2 mg kg^−1^ per injection) were administered intratumorally three times at 3 d intervals. For the laser treatment groups, tumors were irradiated (671 nm, 200 mW, and 100 J) 1 d after each nanoparticle administration (days 8, 11, and 14). Following the final laser treatment, two mice from each group were sacrificed for immediate analysis. The remaining four mice per group were monitored for tumor growth and survival. Tumor dimensions were measured every 2–3 d using digital calipers, and tumor volume was calculated using the formula: (width^2^ × length)/2. Animals were observed until day 21 post‐inoculation and then euthanized in accordance with approved protocols. All animal procedures were conducted in compliance with the guidelines approved by the Institutional Animal Care and Use Committee (IACUC) of SKKU (SKKUIACUC2025‐01‐02‐1).

### In Vivo Histological and Hematological Analyses

On day 21 of the treatment, all mice were sacrificed, and tumors along with major organs were collected for histological and molecular analyses. Major organs were divided into two parts: one fixed in 4% paraformaldehyde (PFA) and the other embedded in OCT compound and snap‐frozen for cryopreservation. Paraffin‐embedded tumor sections were stained with hematoxylin and eosin (H&E) to assess therapeutic efficacy. Stained slides were analyzed using a digital slide scanner (ScanScope CS2, Leica Biosystems). For evaluation of lipid peroxidation and GPX4 expression, immunofluorescence staining for 4‐HNE and GPX4 was performed on paraffin‐embedded tumor sections, followed by imaging via confocal laser scanning microscopy (CLSM). Additionally, intracellular ROS levels were assessed by DCFH‐DA staining of tumor sections obtained from mice sacrificed 1 d after the final laser treatment. These sections were also snap‐frozen in OCT compound before analysis.

### In Vivo Biosafety Assessment

On day 21 post‐treatment, blood samples were collected from tumor‐bearing mice. Serum was isolated by centrifugation at 3,000 rpm for 30 min and analyzed for key biochemical markers, including aspartate aminotransferase (AST), triglycerides, total cholesterol, glucose, creatinine, and blood urea nitrogen (BUN), using an automated clinical chemistry analyzer (DRI‐CHEM NX500VIC, Fujifilm).

### Statistical Analysis

Statistical analyses were conducted using GraphPad Prism version 8 (GraphPad Software, San Diego, CA, USA). Data were presented as mean ± standard deviation (SD) or mean ± standard error of the mean (SEM), as specified. Comparisons among three or more groups were performed using one‐way analysis of variance (ANOVA) followed by Tukey's post hoc test. Exact p‐values are provided in the corresponding figure legends.

## Conflict of Interest

The authors declare no conflict of interest.

## Ethics Approval Statement

All animal experiments were performed in accordance with the guidelines of the Sungkyunkwan University Institutional Animal Care and Use Committee (IACUC).

## Supporting information



Supporting Information

## Data Availability

The data that support the findings of this study are available from the corresponding author upon reasonable request.
