# Supplementary material for: Photosensitizing Lipid Nanoparticles for Ferroptosis‐Enhanced Photodynamic Cancer Therapy via GPX4 Silencing
Source: Adv Healthc Mater. 2025 Nov 12;15(7):e03748. doi: 10.1002/adhm.202503748 (PMC12908211; doi:10.1002/adhm.202503748)
Supplement: Supplementary file 1 — Supporting Information [file ADHM-15-0-s001.docx]

Supporting Information

**Photosensitizing Lipid Nanoparticles for Ferroptosis-Enhanced Photodynamic Cancer Therapy via GPX4 Silencing**

Ga-Hyun Bae, Seungyong Shin, Joo Dong Park, Eun-Young Koh, Seunghyo Ko, Jieun Han, Chun Gown Park, Dong-Hyun Kim, Kun Na, and Wooram Park*

*Corresponding authors

G.-H. Bae, E.-Y. Koh, and Prof. W. Park

Department of MetaBioHealth, Institute for Cross-disciplinary Studies (ICS), Sungkyunkwan University (SKKU), Seobu-ro 2066, Suwon, Gyeonggi 16419, Republic of Korea

E-mail: parkwr@skku.edu (W.P.)

S. Shin, J.D. Park, S. Ko, Dr. J. Han, and Prof. W. Park

Department of Integrative Biotechnology, College of Biotechnology and Bioengineering, SKKU, Seobu-ro 2066, Suwon, Gyeonggi 16419, Republic of Korea

Prof. C.G. Park

Department of Biomedical Engineering, ICS, SKKU, Seobu-ro 2066, Suwon, Gyeonggi 16419, Republic of Korea

Prof. D.-H. Kim

Department of Radiology, Feinberg School of Medicine, Northwestern University, Chicago, IL, 60611, USA

Prof. D.-H. Kim

Robert H. Lurie Comprehensive Cancer Center, Northwestern University, Chicago, IL, 60611, USA

Prof. D.-H. Kim

Department of Biomedical Engineering, McCormick School of Engineering, Northwestern University, Evanston, IL, 60208, USA

Prof. K. Na

Department of Biomedical-Chemical Engineering, The Catholic University of Korea, Jibong-ro 43, Bucheon, Gyeonggi 14662, Republic of Korea

Prof. K. Na

Department of Biotechnology, The Catholic University of Korea, Jibong-ro 43, Bucheon, Gyeonggi 14662, Republic of Korea

**Funding**

This work was supported by the National Research Foundation of Korea (NRF) grant funded by the Korean government (the Ministry of Science & ICT, MSIT) (RS-2024-00350878), by the Bio & Medical Technology Development Program of the NRF funded by the MSIT (RS-2024-00440714), by the Korean Fund for Regenerative Medicine (KFRM) funded by MSIT and the Ministry of Health and Welfare, Republic of Korea (RS-2025-02223118), and by the Korea Basic Science Institute (National Research Facilities and Equipment Center) grant funded by the Korean government (MSIT) (RS-2024-00402899).

**Keywords:** ferroptosis, photodynamic therapy, lipid nanoparticles, GPX4 silencing, cancer nanomedicine

**
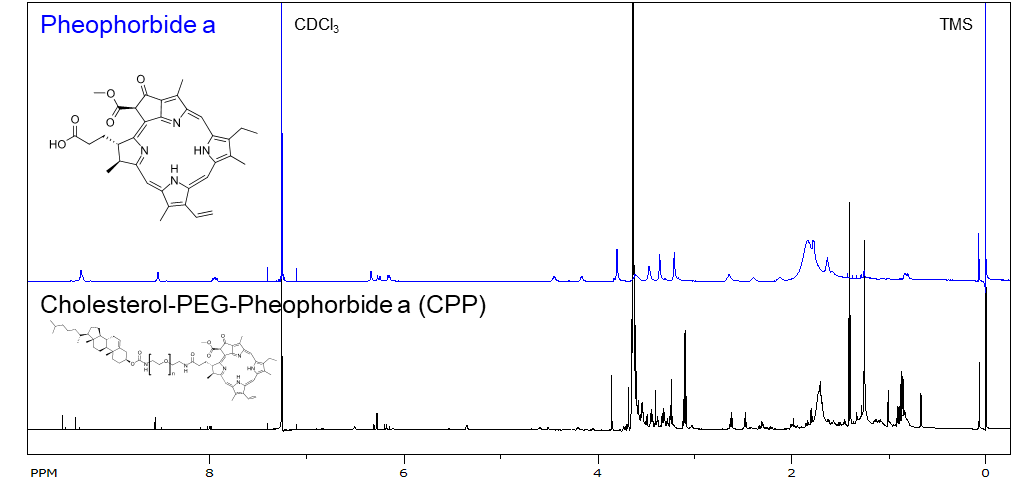
**

**Figure S1.** ¹H NMR (700 MHz, CDCl₃) spectra of pheophorbide a (blue) and cholesterol–PEG–pheophorbide a (CPP, black) in CDCl₃. The characteristic aromatic peaks of pheophorbide a (9.9–7.9 ppm), PEG region (≈3.6 ppm), and cholesterol aliphatic region (1.3–0.6 ppm) confirm successful conjugation.

**
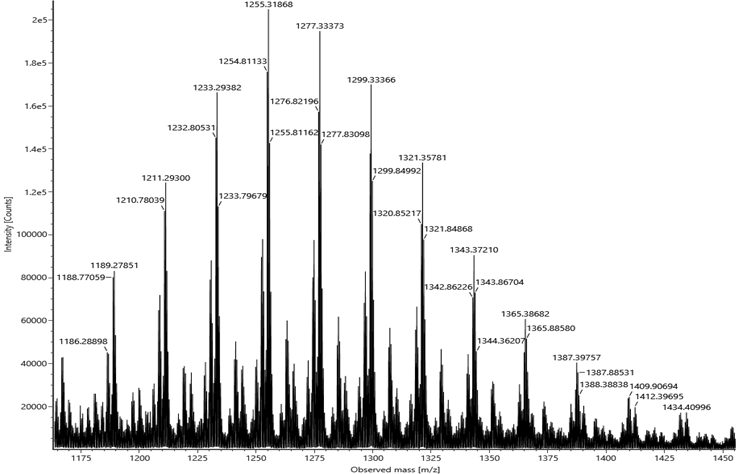
**

**Figure S2.** LC-TOF-MS spectrum of cholesterol–PEG–pheophorbide a conjugate. A regular series of peaks with ~22 m/z spacing was observed, characteristic of PEG repeat units in the doubly charged state. The dominant peak at m/z 1255.3 corresponds to a molecular mass of ~2509 Da, confirming the expected conjugate structure.

**
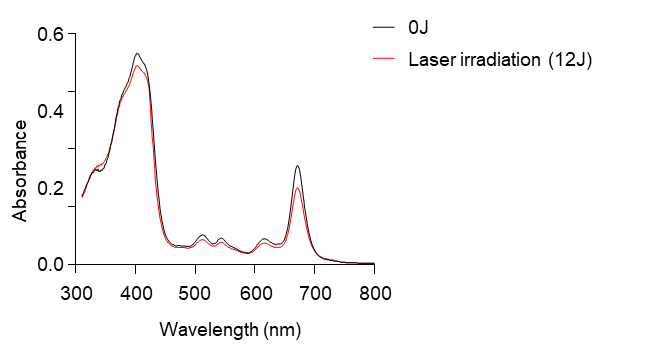
**

**Figure S3.** UV–vis absorption spectra of CPP before and after laser irradiation (12 J) under the same experimental conditions.

**
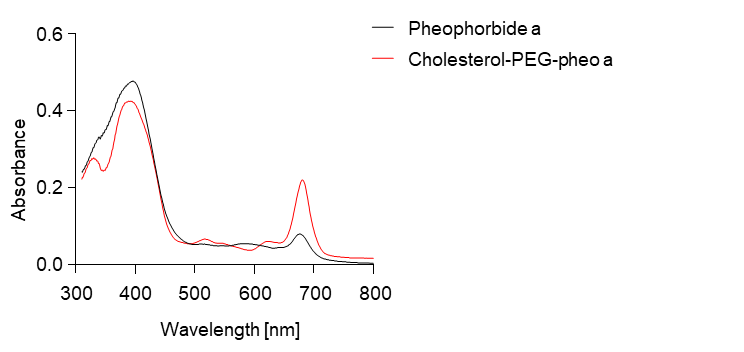
**

**Figure S4.** UV–vis absorption spectra of pheophorbide a and CPP.


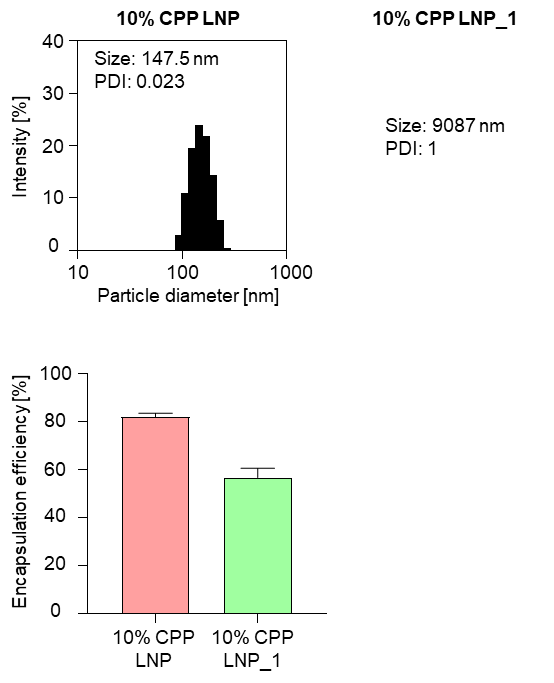


**Figure S5.** Effect of CPP substitution for DMG-PEG2k on the physicochemical properties of LNPs.


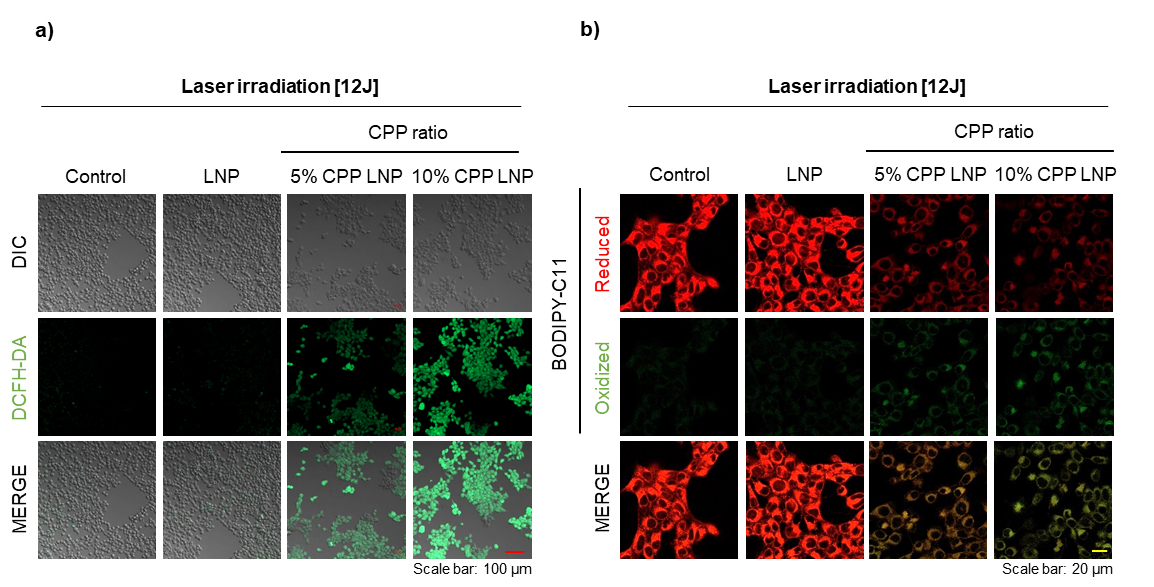


**Figure S6.** Comparison of photodynamic therapy efficacy between 5% and 10% CPP LNPs in 4T1 breast cancer cells. a) Representative CLSM images of 4T1 cells stained with the ROS indicator DCFH-DA (green) after treatment with LNPs or 5% and 10% CPP LNPs followed by laser irradiation. Scale bars: 100 µm. b) Representative CLSM images of 4T1 cells stained with BODIPY-C11 to assess lipid peroxidation following the same treatments. Scale bars: 20 µm.


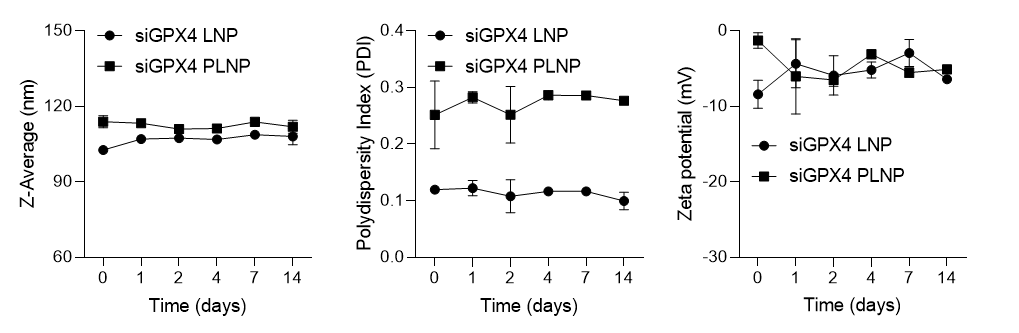


**Figure S7.** Storage stability of siGPX4 LNPs and siGPX4 PLNPs, determined by measuring the hydrodynamic diameter (z-average), polydispersity index (PDI), and zeta potential via DLS over 14 days of storage at 4 °C.


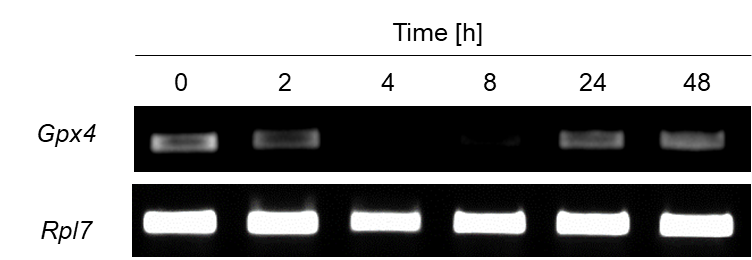


**Figure S8.** RT-PCR analysis of GPX4 mRNA expression in 4T1 cells at various time points after treatment with siGPX4-loaded PLNPs.


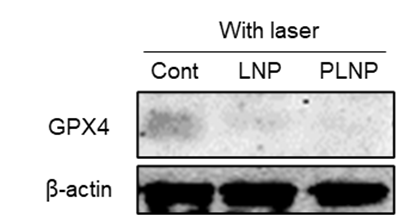


**Figure S9.** Representative images of GPX4 protein expression in 4T1 cells after treatment with LNPs or PLNPs under laser irradiation.


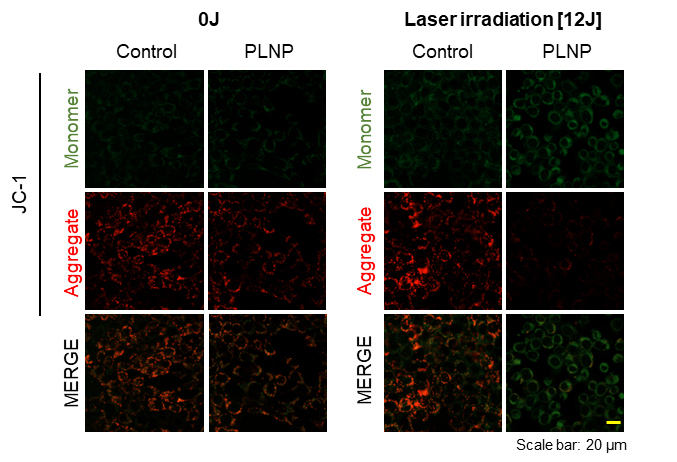


**Figure S10.** Representative CLSM images of JC-1-stained 4T1 cells treated with siGPX4 PLNPs under or without laser irradiation.


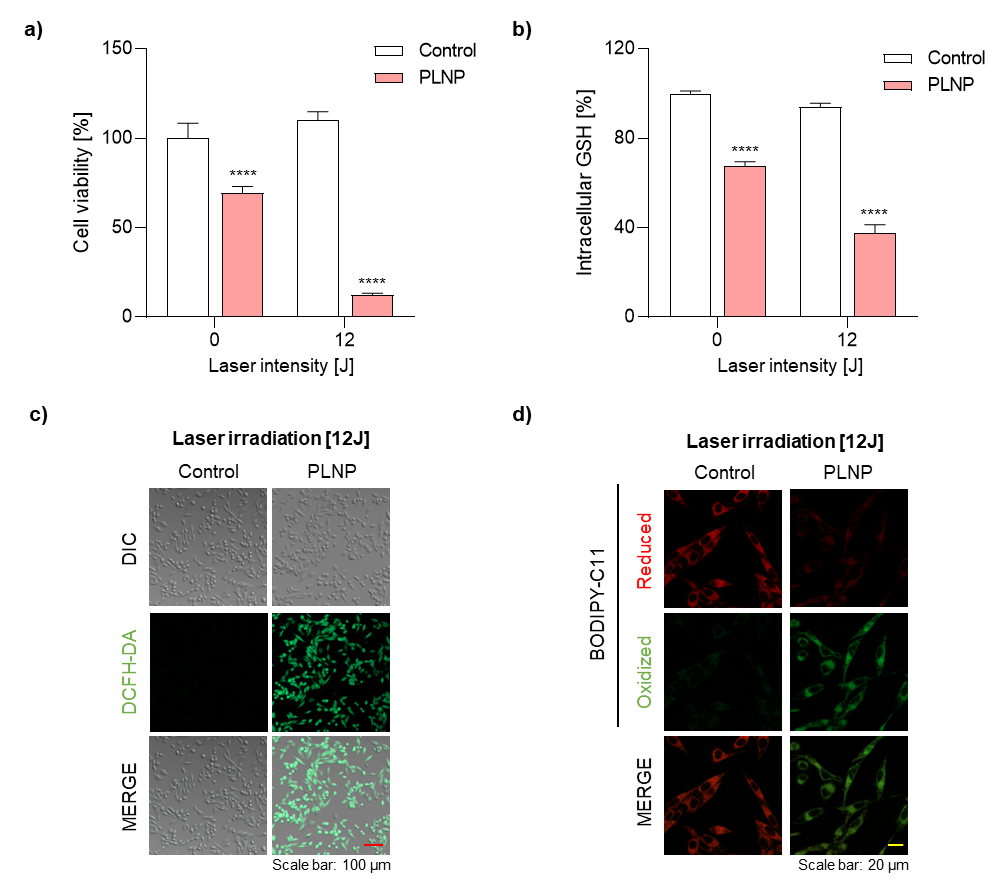


**Figure S11.** *In vitro* evaluation of photodynamic therapy–induced ferroptosis in EO771 breast cancer cells using siGPX4 PLNPs. a) Viability of EO771 cells treated with siGPX4 PLNPs under or without laser irradiation. b) Intracellular GSH levels in EO771 cells after treatment with siGPX4 PLNPs under or without laser irradiation (n = 3). c) Representative CLSM images of EO771 cells stained with the ROS indicator DCFH-DA (green) after treatment with siGPX4 PLNPs under laser irradiation. Scale bars: 100 µm. d) Representative CLSM images of EO771 cells stained with BODIPY-C11 to assess lipid peroxidation after treatment with siGPX4 PLNPs under laser irradiation. Scale bars: 20 µm. Data are presented as the mean ± SD (n = 3). Statistical significance was determined as follows: ns, not significant; *p < 0.05; **p < 0.01; ***p < 0.001; ****p < 0.0001.


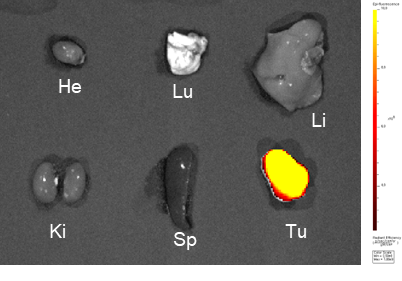


**Figure S12.** Fluorescence imaging showing the distribution of PLNPs in organs harvested from BALB/c mice bearing 4T1 tumors at 48 h post-injection.


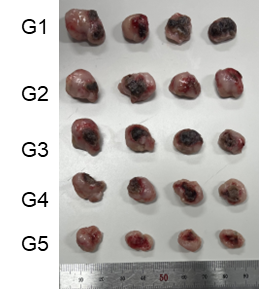


**Figure S13.** Representative photographs of excised tumors on day 21 post-treatment. Group 1 (G1): PBS (control); Group 2 (G2): siNC PLNP; Group 3 (G3): siGPX4 PLNP; Group 4 (G4): siNC PLNP + Laser; Group 5 (G5): siGPX4 PLNP + Laser.


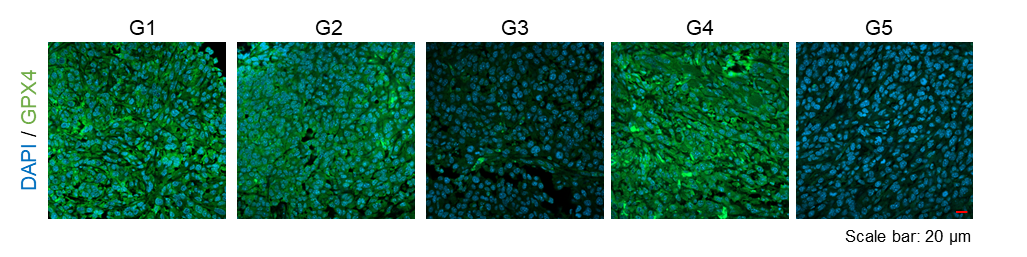


**Figure S14.** Representative CLSM images of tumor sections stained with anti-GPX4 antibody (green). Group 1 (G1): PBS (control); Group 2 (G2): siNC PLNP; Group 3 (G3): siGPX4 PLNP; Group 4 (G4): siNC PLNP + Laser; Group 5 (G5): siGPX4 PLNP + Laser.


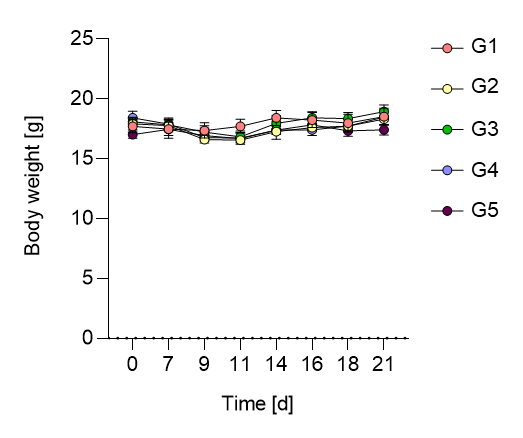


**Figure S15.** Mice body weight monitoring throughout the experimental period. Group 1 (G1): PBS (control); Group 2 (G2): siNC PLNP; Group 3 (G3): siGPX4 PLNP; Group 4 (G4): siNC PLNP + Laser; Group 5 (G5): siGPX4 PLNP + Laser.


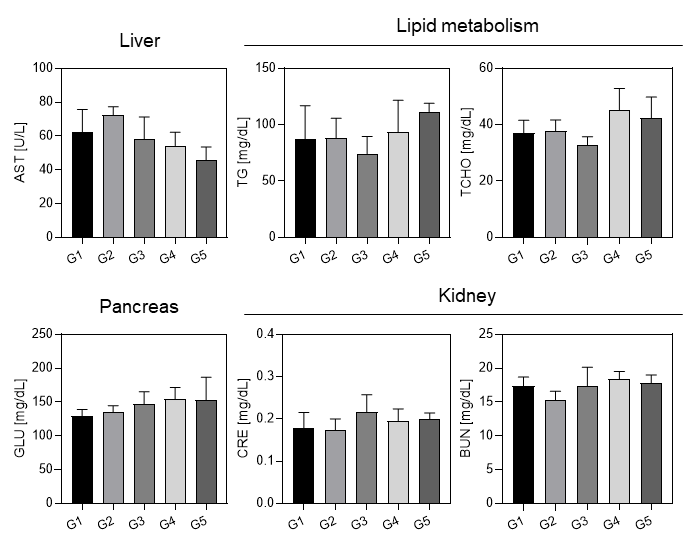


**Figure S16.** Biochemical analysis of plasma parameters following the different therapeutic treatments. Group 1 (G1): PBS (control); Group 2 (G2): siNC PLNP; Group 3 (G3): siGPX4 PLNP; Group 4 (G4): siNC PLNP + Laser; Group 5 (G5): siGPX4 PLNP + Laser.


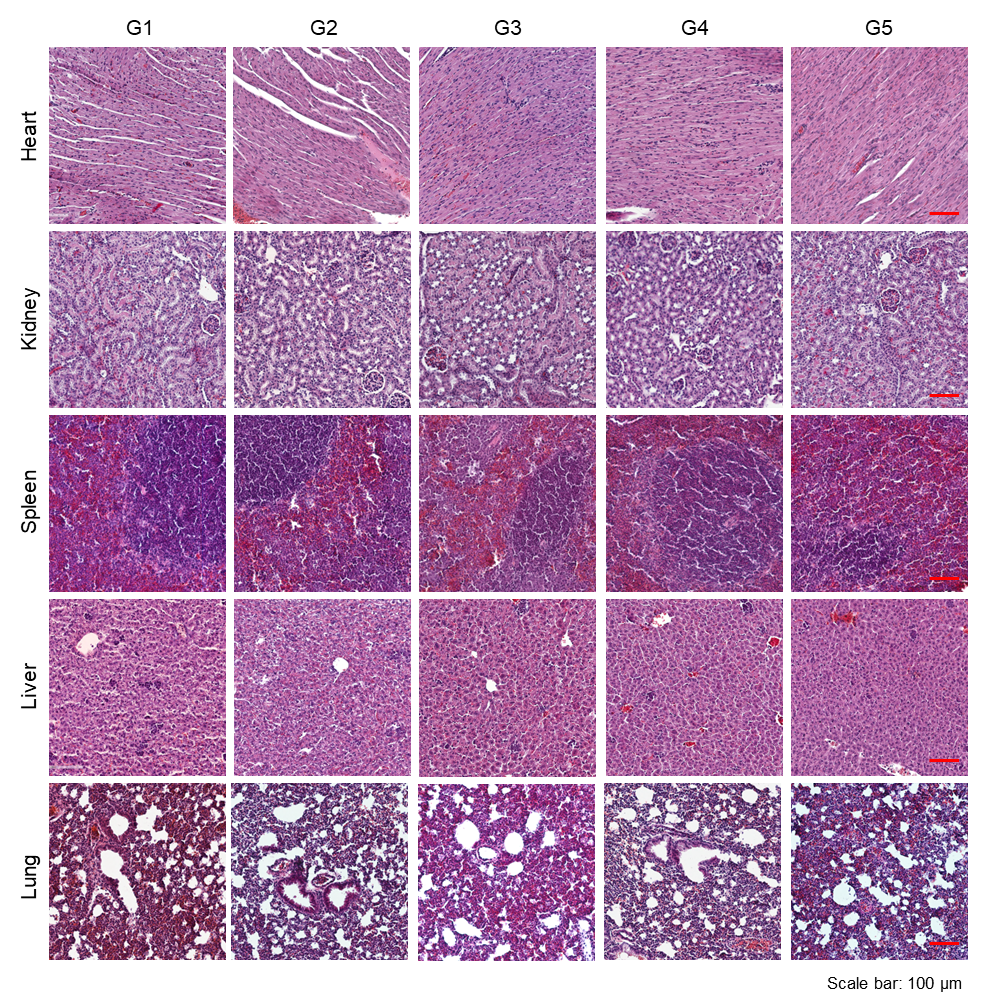


**Figure S17.** H&E staining of major organ sections to assess histopathological changes after the different treatments. Group 1 (G1): PBS (control); Group 2 (G2): siNC PLNP; Group 3 (G3): siGPX4 PLNP; Group 4 (G4): siNC PLNP + Laser; Group 5 (G5): siGPX4 PLNP + Laser.

**Table S1.** Lipid compositions of CPP LNPs formulated with varying CPP molar ratios.

|  | Molar ratio [%] | | | | |
| --- | --- | --- | --- | --- | --- |
| Name | **Ionizable lipid (MC3)^a)^** | **DSPC^b)^** | **Chol-PEG-Pheo a  (CPP)** | **Cholesterol** | **DMG-PEG2k^c)^** |
| LNP | 50 | 10 | 0 | 38.5 | 1.5 |
| 5% CPP LNP | 50 | 10 | 1.925 | 36.57 | 1.5 |
| 10% CPP LNP | 50 | 10 | 3.85 | 34.85 | 1.5 |
| 30% CPP LNP | 50 | 10 | 11.55 | 26.95 | 1.5 |

^a)^DLin–MC3−DMA

^b)^1,2-distearoyl-sn-glycero-3-phosphocholine (DSPC)

^c)^1,2-dimyristoyl-rac-glycero-3-methoxypolyethylene glycol-2000 (DMG-PEG2k)**Table S2.** Lipid compositions of CPP-LNPs formulated with varying CPP molar ratios relative to DMG-PEG2k or cholesterol.

|  | Molar ratio [%] | | | | |
| --- | --- | --- | --- | --- | --- |
| Name | **Ionizable lipid (MC3)^a)^** | **DSPC^b)^** | **Chol-PEG-Pheo a (CPP)** | **Cholesterol** | **DMG-PEG2k^c)^** |
| 10% CPP LNP | 50 | 10 | 3.85 | 34.65 | 1.5 |
| 10% CPP LNP-1 | 50 | 10 | 3.85 | 36.15 | 0 |

^a)^DLin–MC3−DMA

^b)^1,2-distearoyl-sn-glycero-3-phosphocholine (DSPC)

^c)^1,2-dimyristoyl-rac-glycero-3-methoxypolyethylene glycol-2000 (DMG-PEG2k)

**Table S3.** Antibodies used for Western blotting and immunofluorescence staining.

| Antibody | Company | Catalog number |
| --- | --- | --- |
| GPX4 | ABclonal | A25009 |
| GAPDH | ABclonal | AC002 |
| ꞵ-actin | ABclonal | AC004 |
| 4-Hydroxynonenal  (4-HNE) | Bioss | BS-6313R |

**Table S4.** Primer sequences used in RT-PCR analysis.

| Primer | Forward (5’ 🡪 3’) | Reverse (3’ 🡪 5’) |
| --- | --- | --- |
| Mouse RPL7 | TCAATGGAGTAAGCCCAAAG | CAAGAGACCGAGCAATCAAG |
| Mouse GPX4 | CCGATATGCTGAGTGTGGTTTA | GGCTGCAAACTCCTTGATTTC |
